# Design of Multi-DC Overdriving Waveform of Electrowetting Displays for Gray Scale Consistency

**DOI:** 10.3390/mi14030684

**Published:** 2023-03-19

**Authors:** Yijian Xu, Shixiao Li, Ziyang Wang, Heng Zhang, Zikai Li, Bo Xiao, Wei Guo, Linwei Liu, Pengfei Bai

**Affiliations:** Guangdong Provincial Key Laboratory of Optical Information Materials and Technology & Institute of Electronic Paper Displays, South China Academy of Advanced Optoelectronics, South China Normal University, Guangzhou 510006, China

**Keywords:** electrowetting display (EWD), gray scale, overdriving waveform, charge trapping, direct current (DC)

## Abstract

Gray scale consistency in pixels was extremely important for electrowetting displays (EWDs). However, traditional electrowetting display driving waveforms could not obtain a pixel aperture ratio consistency, which led to the occurrence of gray inconsistency even if it was the same driving waveform. In addition, the oil backflow caused by charge trapping could not be sustained. Therefore, a multi-direct current (DC) overdriving waveform for gray scale consistency was proposed in this paper, which could effectively improve the performance of EWDs. The driving waveform was divided into a start-up driving phase and a stable driving phase. The stable driving phase was composed of a square wave with a duty cycle of 79% and a frequency of 43 Hz. Subsequently, an overdriving pulse was also introduced in the stable driving phase. The multi-DC driving waveform for gray scale consistency was applied to a thin film transistor-electrowetting display (TFT-EWD). The average difference between increasing driving voltage and decreasing driving voltage was only 2.79%. The proposed driving waveform has an aperture ratio of 3.7 times at low voltages compared to DC driving.

## 1. Introduction

Electrowetting display (EWD) is a new reflection display technology, boasting high contrast, ultra-low power consumption, and fast response [1,2]. However, the commercialization of EWD still needs to be improved, due to oil backflow problems [3], as well as gray scale consistency and other issues [4]. Driving waveform is a voltage sequence which can drive pixels to display gray scales in EWDs. As one of the key technologies, it directly affects the display effect of pixels; a large number of pixel voltages need to be accurately controlled for playing images and videos [5], and optimization of the driving waveform is one of the important ways to improve the display performance of EWDs [6,7]. A good driving waveform could regulate the direction of movement of oil and display a target gray. Therefore, improving EWD gray scale consistency by optimizing the driving waveform design was efficient.

The EWD technology was proposed by Beni and Hayes [8,9]. In particular, it has attracted a lot of attention in the areas of digital microfluidics (DMF), lab-on-chip, micro-lens, and EWDs [10,11,12,13]. EWD has numerous achievements and applications in the fields of driving waveform [14,15]. However, the charge trapping, contact angle hysteresis, and oil splitting affected the performance of electrowetting displays; therefore, oil backflow, screen flicker, and afterimage may occur [16,17,18]. The aperture ratio of pixels was positively correlated to applied voltages due to the Lippmann–Young equation. But oil could backflow when constant voltage was applied continuously [5]. Oil backflow could be caused by the imbalance between the Laplace pressure and the Maxwell pressure at the three-phase contact line [19]. In recent years, a pulse width modulated (PWM) waveform has been used as the EWD driving waveform [20]. The oil backflow was effectively inhibited by adding a reset signal to the driving waveform, but it could lead to a flicker phenomenon in TFT-EWD displays [21]. It was difficult to guarantee grayscale consistency when displaying gray scale, and gray scale distortion may occur. The gray scale could be precisely controlled by an alternating current (AC) driving waveform based on contact angle hysteresis [17]. The AC driving waveform could effectively suppress the oil backflow and flicker, but the introduction of negative voltage increased the complexity of the circuit design [18]. Overdriving waveforms were widely applied to reduce the hysteresis effect of oil and suppress oil splitting, and achieved a good display effect [22,23].

To suppress the oil backflow generated by charge trapping, a multi-DC overdriving waveform for gray scale consistency was designed. The driving waveform was divided into a start-up driving phase and a stable driving phase. The start-up driving phase waveform was principally used to turn on the oil rapidly while the stable driving phase waveform was principally used to preserve the stability of the aperture ratio. The problems caused by charge trapping could be effectively avoided by the driving waveform so that the EWD has a good consistency and stability when switching gray scales.

## 2. Principle of EWDs

### 2.1. Principle of EWDs

**Figure 1:**
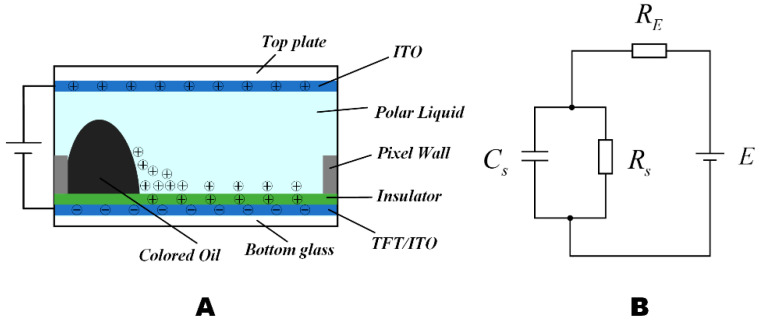
The EWD structure and its simplified equivalent circuit diagram. (**A**) Side view of EWD pixel structure under an electric field. (**B**) A simplified equivalent RC circuit model of EWD pixels.

An EWD changed pixels in the wettability of polar liquids in an insulating hydrophobic layer by applying a driving voltage between the upper and lower indium tin oxide (ITO) electrodes, resulting in change and displacement [24]. Each pixel of EWDs was principally comprised of a top plate, ITO/TFT glass, polar liquid, color oil, pixel wall, a hydrophobic insulating layer, and substrates. As shown in Figure 1A, when a potential difference was applied between the liquid and the electrode, the electric field force acts on ions in the liquid and pulls them towards the insulating layer. When the interaction between the ion and the electrode is stronger than that between the ion and the liquid, the charge is likely to be captured in the insulating layer. Charged droplets or clusters of molecules can move into the nanopores of the insulating layer and be captured [25]. This is the principle of charge trapping. The oil backflow is caused by charge trapping, resulting in afterimages on EWDs, and the continuous display of the image cannot be maintained, with the consequence that target gray scales cannot be displayed. The overall equivalent circuit model of a pixel can be defined as an RC (resistance–capacitance) circuit; as a capacitor, the display effect of pixels is related to the capacitance, as shown in Figure 1B [26].

(1) $$C=\frac{1}{4\pi k}(\frac{\varepsilon_{oil}S_{oil}}{h}+\frac{\varepsilon_{r}S_{pix}}{d})$$

(2) $$f1=\frac{1}{2\pi R_{s}C_{s}}$$

(3) $$f2=\frac{1}{2\pi C_{s}(\frac{R_{E}R_{S}}{R_{E}+R_{S}})}$$

The capacitance value of pixels can be calculated by Equation (1) [27] where k is the electrostatic constant, h is the thickness of the oil, $\varepsilon_{r}$ represents the dielectric constant of the hydrophobic insulating layer, $\varepsilon_{oil}$ is the dielectric constant of oil, $S_{oil}$ is the area of oil which shrinks to the corners of a pixel, $S_{pix}$ represents the area of a pixel, and d is the thickness of the hydrophobic insulating layer. The RC series-parallel circuit possesses two mutation frequencies f1 and f2, and the calculation formulas are shown in Equations (2) and (3) [28,29,30] where E is the driving voltage, $C_{S}$ is the capacitance of the EWD pixel, $R_{S}$ is the resistance of the EWD pixel, and $R_{E}$ is the external resistance under the action of an external driving voltage. When the capacitance of $C_{S}$ is a constant, the mutation frequencies f1 and f2 in the circuit can reach a steady value.

(4) $$\sigma_{L}=\frac{\varepsilon_{0}\varepsilon_{r}(V-V_{T})}{d}$$

(5) $$\gamma_{LV}\left[cos\theta_{V}-cos\theta_{0}\right]=\frac{1}{2}\frac{\varepsilon_{0}\varepsilon_{r}}{d}{\left(V-V_{T}\right)}^{2}$$

(6) $$A=[1\;-\frac{S_{oil}}{S_{pix}}]\times 100\%$$

When a constant voltage is applied, due to charge trapping, the contracted oil cannot be retained in a stable state, and the charge is deintercalated into the insulating layer by an electric field force. The charge density in the liquid phase can be calculated by Equation (4) [25] where $\sigma_{L}$ is the charge density in the liquid phase, $\varepsilon_{0}$ is the dielectric constant in the vacuum, and $V_{T}$ is the potential generated by charge trapping. The charge trapping can be compensated by the polarity driving scheme. By changing the polarity of the voltage, the charge can be captured, released, and recaptured in the dielectric layer. It has been experimentally demonstrated that the trapped charge could be released when the applied voltage was diminished, or a specific voltage waveform was applied [31,32,33]. According to Young’s equation shown in Equation (5), the driving voltage and the contact angle are positively correlated [25]. Where $\theta_{V}$ is the contact angle of the solid and the liquid with a certain voltage, $\theta_{0}$ is the contact angle of the solid and the liquid without voltage, and $\gamma_{LV}$ is the free energy of the liquid/vapor interface without an electric field. Electrowetting could also be used to form the basis of a reflective display that was significantly faster than electrophoretic displays [34]. When the driving voltage is applied, different periodical features are found for the droplet dancing at the resonance frequencies and departure from resonance. Though an alternating current voltage would indeed slightly reduce the hysteresis effect, the electrowetting effect can be enhanced by lowering the actuation frequency [35,36,37]. As one of the important indicators of EWDs, the aperture ratio could be directly used to evaluate the effect of the display, as shown in Equation (6) [38] where A is the aperture ratio and the different aperture ratios show different gray scales.

### 2.2. Multi-DC Driving Waveform

**Figure 2:**
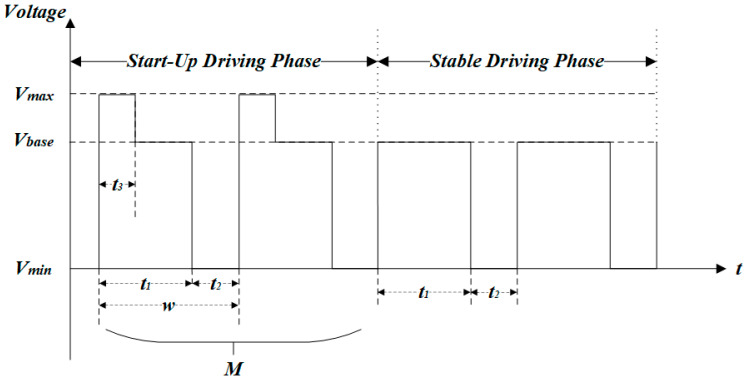
The proposed multi-DC driving waveform.

The multi-DC driving waveform for gray scale consistency is composed of a start-up driving phase and a stable driving phase. As shown in Figure 2, the stable driving phase is constituted of a square waveform with a period *w*. The start-up driving phase consists of a number of M start-up driving waveforms where $t_{3}$ is a driving time of the overdriving pulse, $V_{max}$ is the max driving voltage, and $V_{base}$ is the effective driving voltage. w is the period of a waveform, which is composed of the effective driving time $t_{1}$ and the charge deintercalation time $t_{2}$. The pixel could be switched on by voltage driving at $t_{1}$ and release charges in the insulation layer at $t_{2}$ to obtain a stable gray scale. This is inspired by Zeng [27], Long [39], and Chiu [23], who decrease the pixel response time and inhibit the oil splitting of EWD based on an overdriving waveform; however, the gain effect of each parameter of the overdriving waveform was not described in detail. Therefore, the related characteristics of our proposed driving waveform need to be further studied.

### 2.3. Multi-DC Driving Waveform Applied on the TFT-EWD

**Figure 3:**
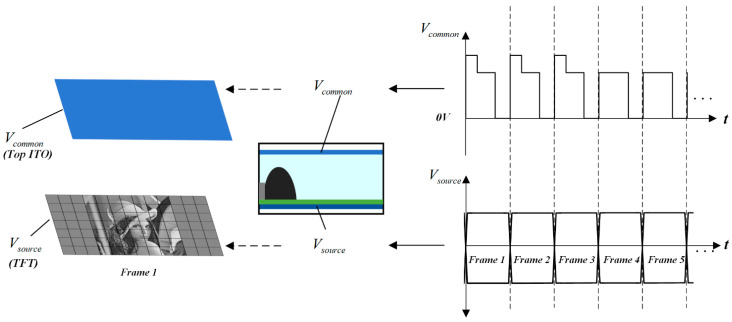
Schematic diagram of multi-DC driving waveform applied on TFT-EWD.

The proposed driving waveform was applied to the TFT-EWD, as shown in Figure 3. A voltage signal was provided by $V_{common}$ for the common pole of the TFT-EWD. $V_{source}$ was the voltage signal of image pixels in the TFT panel. The image could be displayed on the TFT-EWD by the combination of $V_{common}$ and $V_{source}$. Compared with the ITO-EWD, a source driver integrated circuit chip (IC) and a gate driver IC from UltraChip corporation in Taipei, Taiwan, and TFT structure could be used at the TFT-EWD to lock the pixel voltage, in order that the pixel capacitance value could be changed. The proposed driving waveform was adjusted by the structural changes in this paper. As shown in Figure 3, in the start-up driving phase and the stable driving phase, the waveform of each period corresponds to a frame of an image, in order that the low-gray image could be displayed. The details of the image could be displayed by this method.

## 3. Results and Discussion

### 3.1. Experiment System

**Figure 4:**
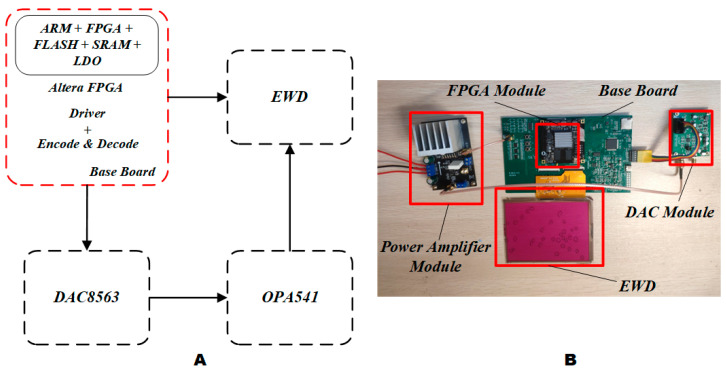
EWD driving waveform experiment system. (**A**) EWD driving waveform experiment system structure diagram. (**B**) EWD driving waveform experiment system diagram.

To evaluate the effectiveness of the driving waveform, an EWD driving waveform experiment system was designed. As shown in Figure 4, the system was composed of a field programmable gate array (FPGA) module, a base board, a digital-to-analog conversion (DAC) module, and a power amplifier module. Altera’s EP4CE75F23C8 was selected as the core control chip of the EWD driver. A DAC8563 was used as a digital-to-analog conversion module to generate an arbitrary driving waveform. An OPA541 operational amplifier was used as a power amplifier module for signal amplification. The signal interface between the various modules was provided and connected by the base board.

**Figure 5:**
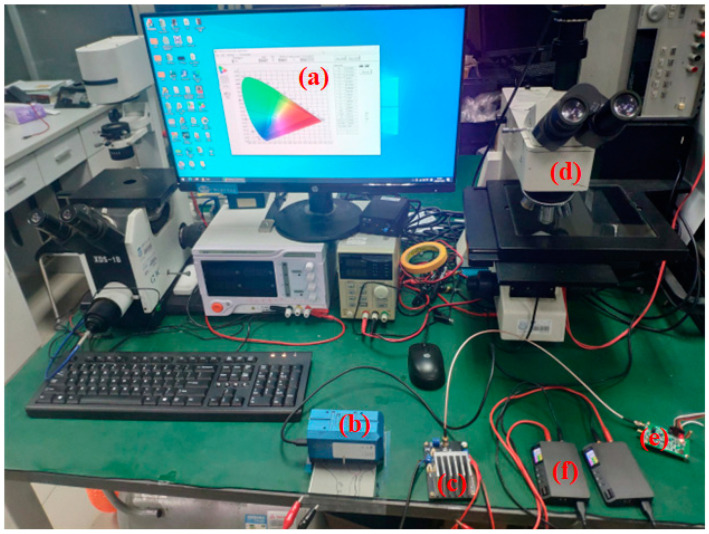
An experimental platform for driving EWDs. (**a**) A computer. (**b**) A colorimeter. (**c**) A power amplifier module. (**d**) A microscope. (**e**) A digital-to-analog conversion module. (**f**) Power supply units.

In order to evaluate the validity of the driving waveform, two experimental platforms were built. Figure 5 was a luminance and aperture ratio testing platform. (a) was a computer for receiving and processing data. (b) was a colorimeter for detecting EWDs’ reflected luminance. (c) was a power amplifier module for signal amplification. (d) was a microscope for observing the EWD’s pixel structure. (e) was a digital-to-analog conversion module for EWD driver control signal conversion. (f) were power supply units. All experiments were conducted at room temperature of 25 °C and 53% humidity.

In sample manufacturing, inkjet printing technology was applied to prepare the insulation layer, followed by a photoetching method to prepare the pixel wall, and inkjet printing technology was applied to fill the oil [40]. As shown in Table 1, where ITO/TFT in the preparation process need to use plasma etcher for etching, the power was 100 W per 100 s. The hydrophobic layer was restored to hydrophobic by reflux at 210 °C for 2 h. Pixels were filled with 18% black oil. The height of the pixel wall was 3.5 um. The aperture ratio of ITO-EWD could reach 61.48% with +30 V DC driving.

**Figure 6:**
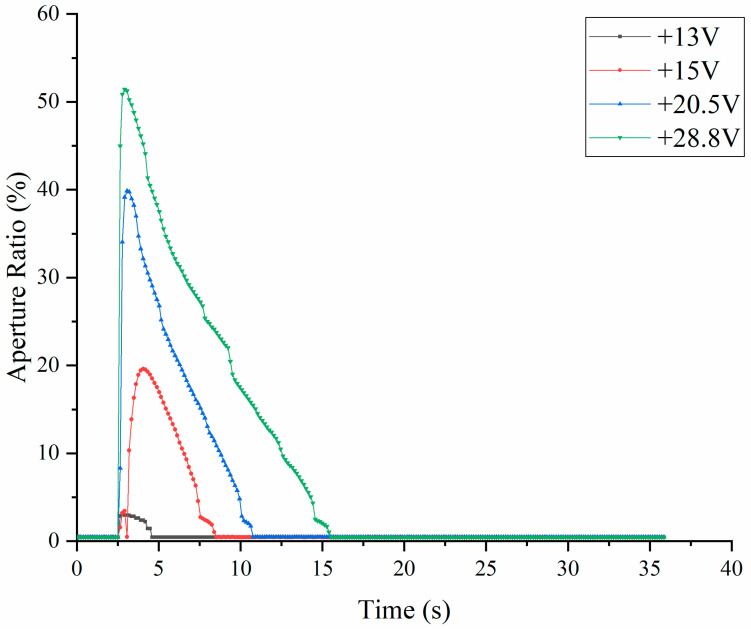
Aperture ratio with a DC voltage.

To understand EWD aperture ratio characteristics, the EWD was tested with a single DC voltage in this paper. As shown in Figure 6, +13 V, +15 V, +20.5 V, and +28.8 V DC voltages were continuously accessed to the EWD for experiments, and the maximum aperture ratio was 51.43% when a +28.8 V single DC voltage driving waveform was applied. The higher the DC voltage, the longer was the backflow time. The oil could turn over the pixel wall when the voltage exceeded +30 V; therefore, in order to ensure that the pixel works, the driving voltage must be below 30 V.

### 3.2. Stable Driving Phase Waveform

**Figure 7:**
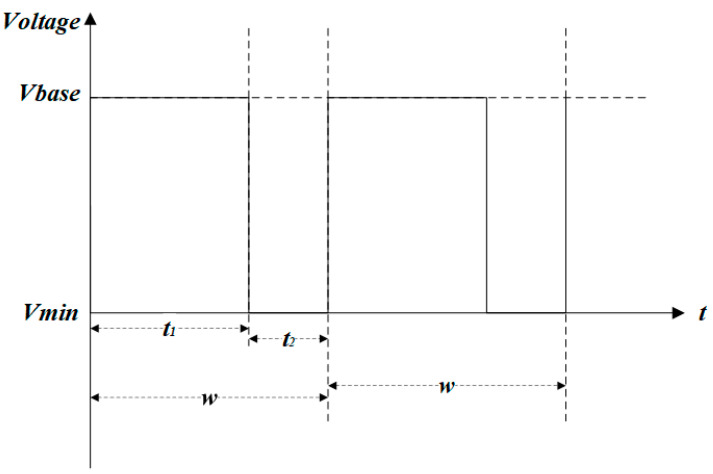
Driving waveforms diagram in a stable driving phase.

The stable driving phase waveform was used to display a consistent gray scale. As shown in Figure 7, a stable driving phase waveform was determined by parameters driving the waveform period (w), the effective driving time ($t_{1}$), the charge deintercalation time ($t_{2}$), the effective driving voltage ($V_{base}$), and the charge deintercalation voltage ($V_{min}$). These parameters would be discussed and analyzed in this paper. When the pixel was in an “on” state, appropriate parameters had to be found to ensure that the pixel aperture ratio remained stable. When a constant voltage was applied, the electrowetting force decreased due to charge trapping [24]. It has been experimentally demonstrated that the trapped charge was released when the applied voltage was lowered [25,33]. Therefore, $V_{min}$ could be set to 0 V to release the captured charge as a DC driving waveform.

#### 3.2.1. Driving Waveform Period (w)

**Figure 8:**
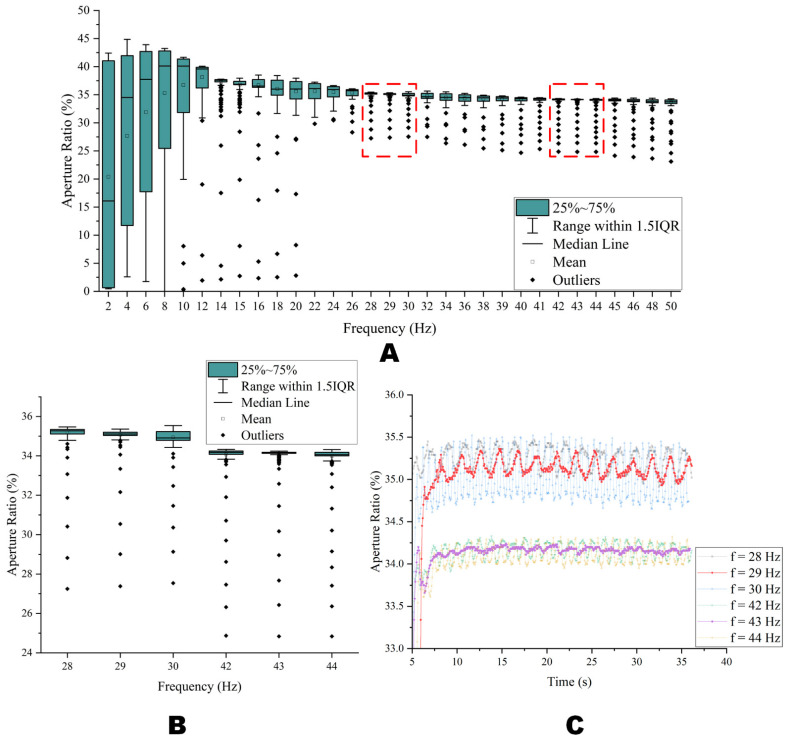
Fluctuations in EWD aperture ratio at different frequencies. (**A**) The EWD aperture ratio box diagram when $V_{base}$ = 20.5 V, $t_{2}$ = 11.63 ms, and the frequency was from 2 Hz to 50 Hz. (**B**) The aperture ratio box diagram at 28 Hz, 29 Hz, 30 Hz, 42 Hz, 43 Hz and 44 Hz when $V_{base}$ = 20.5 V, $t_{2}$ = 11.63 ms. (**C**) The aperture ratio and time relationship diagram at 28 Hz, 29 Hz, 30 Hz, 42 Hz, 43 Hz, and 44 Hz when $V_{base}$ = 20.5 V, $t_{2}$ = 11.63 ms.

Because it is one of the important parameters of the driving waveform, the longer the period, the lower is the power consumption of the EWD drive system. A changed frequency was adopted by this paper for testing. $V_{base}$, $V_{min}$ and the duty cycle were set to +20.5 V, 0 V and 50%, respectively. To obtain the minimum driving waveform frequency under the stable aperture ratio, the changing trend of the aperture ratio was observed by changing the frequency. From 2 Hz to 50 Hz, a test time of 35 s at an interval of 2 Hz was performed to obtain fluctuations in the aperture ratio curve. As shown in Figure 8, the fluctuation in the aperture ratio data was converted to a box diagram; a smaller 25~75% box was obtained in the range of 28 Hz~30 Hz and 42 Hz~44 Hz.

To better observe the fluctuation trend, 29 Hz, 39 Hz, 41 Hz, 43 Hz, and 45 Hz were added for evaluation. Figure 8B was obtained by enlarging the aperture ratio intervals at frequencies of 28 Hz~30 Hz and 42 Hz~44 Hz. Figure 8C shows the relationship between the aperture ratio and time at the frequencies of 28 Hz~30 Hz and 42 Hz~44 Hz. It could be further seen that a more stable aperture ratio could be obtained at 43 Hz, and the occurrence of the oil backflow could be inhibited at this frequency.

As shown in Figure 6, the aperture ratio with a +20.5 V DC driving waveform was 39.84%. When $V_{base}$ was +20.5 V and the frequency was 43 Hz (w = 23.26 ms), the aperture ratio was only decreased by 5.73% compared with the +20.5 V DC driving waveform. However, the 43 Hz square waveform possesses a more stable aperture ratio than the +20.5 V DC driving waveform. Therefore, in this paper, 23.26 ms was the longest driving waveform period when the aperture ratio was stable. The 43 Hz was the lowest frequency to ensure the stability of the aperture ratio, which could be used in this paper.

#### 3.2.2. Charge Deintercalation Time ($t_{2}$)

**Figure 9:**
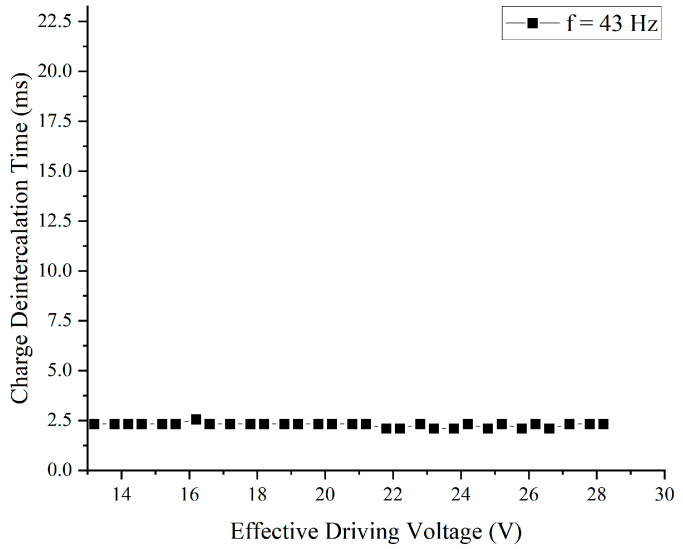
The minimum charge deintercalation time with diverse driving voltage ($V_{base}$).

When using 43 Hz as the frequency of the waveform, $t_{2}$ needs to be determined to stabilize the display of the pixel. In this paper, $t_{2}$ was defined as the deintercalation time, releasing the charge deintercalation in the insulation layer. To further study the relationship between $V_{base}$ and $t_{2}$, an ITO-EWD was used for testing, and the minimum charge deintercalation times $t_{2}$ under different $V_{base}$ voltages were tested respectively. By observing EWD pixels driven by $V_{base}$, $t_{2}$ gradually increased from 0 ms until the pixel aperture ratio reached the maximum value, and no oil backflow occurred. At this point, $t_{2}$ was the minimum charge deintercalation time at $V_{base}$. As shown in Figure 9, when the frequency was 43 Hz and the $V_{min}$ voltage was 0 V, the minimum charge deintercalation time was independent of the $V_{base}$ and tended to be a constant. The average minimum charge deintercalation time of the ITO-EWD was 2.28 ms. Therefore, 2.28 ms was defined as the minimum charge deintercalation time of the ITO-EWD. To enhance the stability and compatibility of the driving waveform, the driving waveform increased by more than 10% of the cycle as the $t_{2}$ margin of the deintercalation time; therefore, 4.88 ms was used as the stable deintercalation time.

#### 3.2.3. Effective Driving Voltage ($V_{base}$)

**Figure 10:**
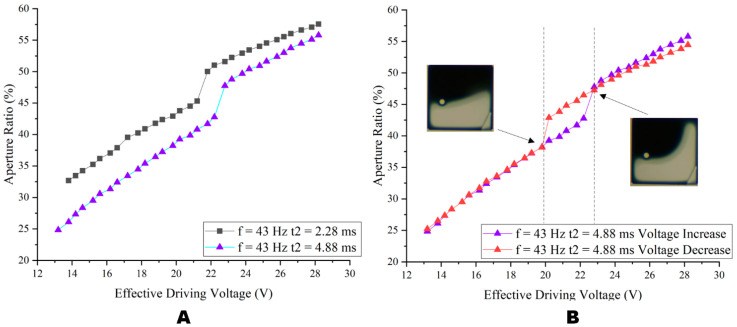
The relationship between the $V_{base}$ and aperture ratio. (**A**) The aperture ratio of the ITO-EWD was tested when the $V_{base}$ increased from 13 V to 28.8 V. (**B**) The aperture ratio of the ITO-EWD was tested when the voltage increased from 13 V to 28.8 V and decreased from 28.8 V to 13 V.

Multi-level gray scales could be obtained by changing the $V_{base}$. To further investigate the effect of the $V_{base}$ on the aperture ratio, the aperture ratio of the ITO-EWD was tested with a different $V_{base}$. As shown in Figure 10A, the change in aperture ratio of charge deintercalation at 2.28 ms and 4.88 ms, respectively. When the voltage was increased from 13 V to 28.8 V, the aperture ratio of the ITO-EWD showed a linear increase trend. With the same $V_{base}$, the aperture ratio of the ITO-EWD at $t_{2}$ = 2.28 ms was 4.56% higher than that at $t_{2}$ = 4.88 ms on average.

When the $V_{base}$ voltage was between 19.8 V and 22.8 V, there was an obvious aperture ratio jump, which was believed to be related to the pixel structure. As shown in Figure 10B, because the pixel was applied voltage in the lower right triangle area, the area near the electrode was first opened so that oil was squeezed above the pixel. When a higher voltage was applied, the opening direction increased at the top right instead of pushing up again, resulting in a sudden change in the aperture ratio.

To test the effect of the driving voltage on the consistency of the aperture ratio, the aperture ratios of pixels with the $V_{base}$ increasing from 13 V to 28.8 V and decreasing from 28.8 V to 13 V were compared. As shown in Figure 10B, when the $V_{base}$ increases from 13 V to 28.8 V and the $V_{base}$ decreases from 28.8 V to 13 V, the aperture ratio of the ITO-EWD is almost the same. The average aperture ratio difference between the voltage increase and the voltage decrease was only 2.79% in the ITO-EWD. In the stable driving stage, the aperture ratio has a high consistency. As could be seen above, the aperture ratio presents a linear change by changing the driving voltage, and this method ensures the consistency of the aperture ratio. Therefore, gray scale adjustment was realized by changing the effective driving voltage in this paper. Furthermore, by regulating the $V_{base}$, f, and $t_{2}$, a better aperture ratio could be obtained.

### 3.3. Start-Up Driving Phase Waveform

**Figure 11:**
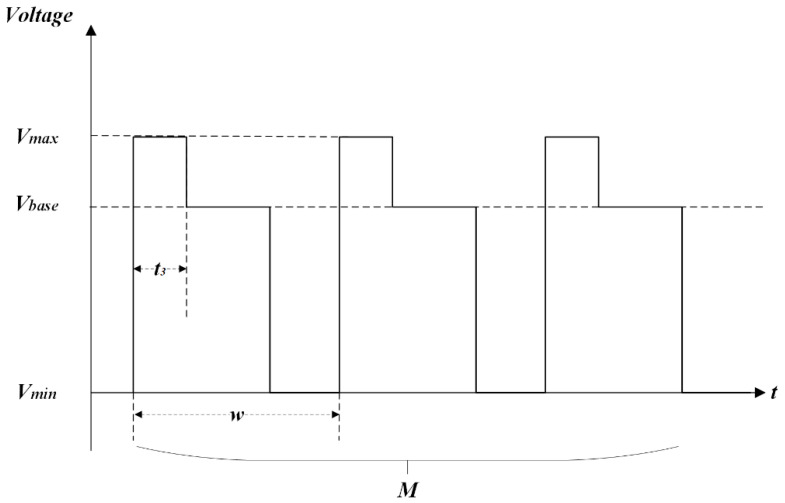
Driving waveform diagram in the start-up driving phase.

The EWD could display different gray scales and avoid the oil backflow effectively by a stable drive phase waveform. In addition, to open the EWD pixel rapidly and increase the adjustable range of the gray scale, the overdriving pulse was introduced. As shown in Figure 11, next, the influence of the overdriving pulse voltage ($V_{max}$), the overdriving time ($t_{3}$), and the number of overdriving waveforms (M) will be further discussed.

In order to reduce the switch on time and avoid the influence of the overdriving pulse on the aperture ratio of the driving waveform in the stable driving phase, the overdriving pulse waveform parameters were studied in this paper.

**Figure 12:**
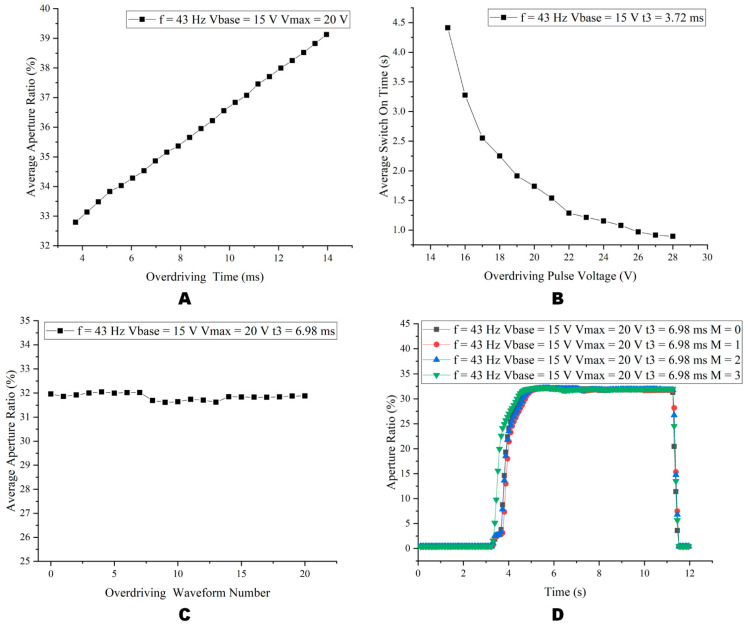
Influence of parameters of the overdriving waveform on the aperture ratio. (**A**) When f = 43 Hz, $V_{base}$ = 15 V, $V_{max}$ = 20 V, the relationship between $t_{3}$ and aperture ratio. (**B**) When f = 43 Hz, $V_{base}$ = 15 V, $t_{3}$ = 3.72 ms, the relationship between $V_{max}$ and average switch on time. (**C**) When f = 43 Hz, $V_{base}$ = 15 V, $V_{max}$ = 20 V, $t_{3}$ = 3.72 ms, the relationship between M and aperture ratio. (**D**) When f = 43 Hz, $V_{base}$ = 15 V, $V_{max}$ = 20 V, $t_{3}$ = 6.98 ms, the relationship between time and aperture ratio for different numbers of waveforms.

As shown in Figure 12, relevant parameters of the overdriving waveform were tested. In the experiment, the FPGA outputs a 10-s driving waveform to open the pixel, and records the average aperture ratio after the pixel was opened and stabilized. Then, a 0 V was output for 10 s to close the pixel. The preceding steps were repeated 10 times. The average value of 10 experimental data was used as the average aperture ratio. It could be seen from Figure 12A that the overdriving time $t_{3}$ was proportional to the average aperture ratio. When the EWD worked at f = 43 Hz, $V_{base}$ = 15 V, $V_{max}$ = 20 V, and $t_{3}$ = 3.72 ms, the lowest aperture ratio of the pixel was obtained. As shown in Figure 12B, $V_{max}$ was inversely proportional to the average switch on time of the pixel, and a minimum of 28.8 V. To study the influence of the number of overdriving waveforms on the aperture ratio, it could be seen from Figure 12C that as the number of overdriving waveforms increased, the maximum average aperture ratio was 32.04%, and the minimum average aperture ratio was 31.61%. The difference between the maximum and minimum aperture ratio was only 0.43%, indicating that the number of overdriving waveforms had negligible influence on the aperture ratio. According to Figure 12D, the pixel switch on time was faster and the opening rate remained stable when M = 3. Therefore, the start-up driving phase waveform could effectively reduce the pixel switch on time and stabilize the aperture ratio. We used $t_{3}$ = 3.72 ms, $t_{2}$ = 4.88 ms, $V_{max}$ = 28.8 V, M = 3, and f = 43 Hz as the multi-DC driving waveform parameters of the switching gray scale experiment.

**Figure 13:**
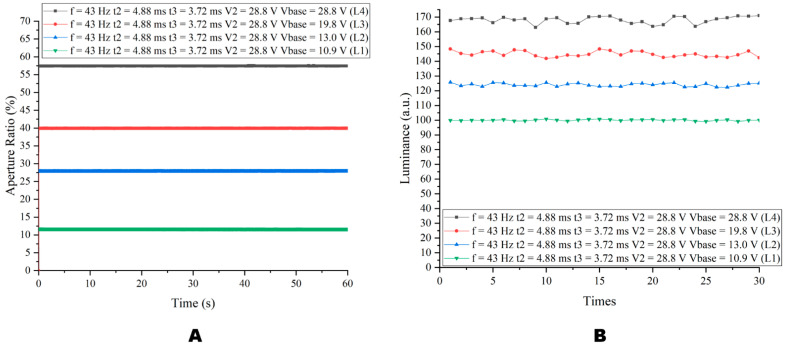
Aperture ratio and luminance at four $V_{base}$ levels. In the case of f = 43 Hz, $t_{2}$ = 4.88 ms, $t_{3}$ = 3.72 ms, and $V_{max}$ = 28.8 V, four levels of gray scales could be achieved by changing $V_{base}$. (**A**) Aperture ratio at four $V_{base}$. (**B**) Luminance at four $V_{base}$.

We displayed four levels of gray scales with the multi-DC driving waveform proposed in this paper, as shown in Figure 13. Based on the above experiments, when f = 43 Hz, $t_{2}$ = 4.88 ms, $t_{3}$ = 3.72 ms, and $V_{max}$ = 28.8 V, only the $V_{base}$ could be modified to achieve a gray scale adjustment. $V_{base}$ voltages corresponding to L1, L2, L3 and L4 were 10.9 V, 13 V, 19.8 V and 28.8 V, respectively. As shown in Figure 13A, the aperture ratio corresponded to four $V_{base}$ voltages. The aperture ratios corresponding to L1 and L4 gray scales were 11.28% and 56.13%, respectively. Figure 13B shows the reflected luminance corresponding to four $V_{base}$ voltages. The minimum reflected luminance was 96.74 absorbance units (A.U.) and the maximum reflected luminance was 170.5 (A.U.). It could be seen from Figure 13B that level 4 reflected luminance was distributed uniformly.

**Figure 14:**
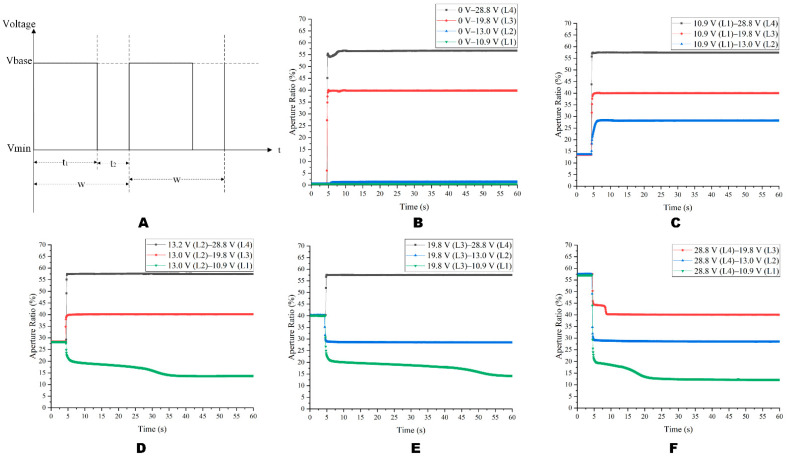
A change in switching between four gray scale levels driven by a square wave. (**A**) The square wave driving waveform experiment diagram. (**B**) The curve of the aperture ratio when $V_{base}$ changed from 0 V to L1~L4. (**C**) The curve of the aperture ratio when $V_{base}$ changed from L1 to L2~L4. (**D**) The curve of the aperture ratio when $V_{base}$ changed from L2 to L1, L3, and L4. (**E**) The curve of the aperture ratio when $V_{base}$ changed from L3 to L1, L2, and L4. (**F**) The curve of the aperture ratio when $V_{base}$ changed from L4 to L1~L3.

Figure 14 shows the change in the aperture ratio when switching the gray scale under a square wave. Figure 14A shows the square wave used in the experiment, and its relevant parameters were w = 23.26 ms (f = 43 Hz), $t_{2}$ = 4.88 ms, and $t_{1}$ = 18.38 ms. As could be seen in Figure 14B, the voltage from 0 V to the L1 and L2 gray scale could not penetrate the oil and the pixels could not be opened. It could be seen from Figure 14 that when the gray scale decreased from the high gray scale to the L1 gray scale, it took a long time to achieve a stable aperture ratio.

**Figure 15:**
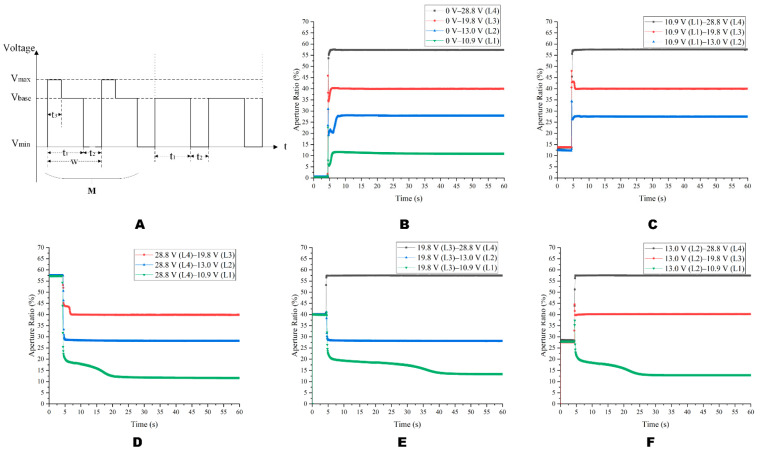
The diagram of the aperture ratio switching between four gray scales under the multi-DC driving waveform. (**A**) The multi-DC driving waveform diagram for gray scale consistency. (**B**) The curve of the aperture ratio when $V_{base}$ changed from 0 V to L1~L4. (**C**) The curve of the aperture ratio when the voltage of the $V_{base}$ changed from L1 to L2~L4. (**D**) The curve of the aperture ratio when the $V_{base}$ changed from L2 to L1, L3, and L4. (**E**) The curve of the aperture ratio when the $V_{base}$ changed from L3 to L1, L2, and L4. (**F**) The curve of the aperture ratio when the $V_{base}$ changed from L4 to L1~L3.

Based on the stable driving phase, the overdriving pulse was added, and the driving waveform is shown in Figure 15A. The relevant parameters were w = 23.26 ms (f = 43 Hz), $t_{2}$ = 4.88 ms, $t_{3}$ = 3.72 ms, $V_{max}$ = 28.8 V and M = 6. As shown in Figure 15B, different from the square wave, the driving waveform proposed in this paper successfully made the EWD generate four gray scales from the 0 V. As could be seen from Figure 14D,E and Figure 15D,E, proposed waveforms have good aperture ratio consistency and a faster response time. From L2 to L1, the proposed waveform was 14.57 s faster than the square wave, and the gray scales could be stabilized faster. As could be seen from Figure 6 and Figure 15B, the driving waveform proposed in this paper could make the aperture ratio with 13 V driving reach 11.31%, which was 3.7 times that of the DC driving. When the driving voltage was 13 V, the aperture ratio of the driving waveform proposed in this paper reached 11.31%, which was 3.7 times that of the DC driving.

### 3.4. Multi-DC Driving Waveform Applied to TFT-EWD

**Figure 16:**
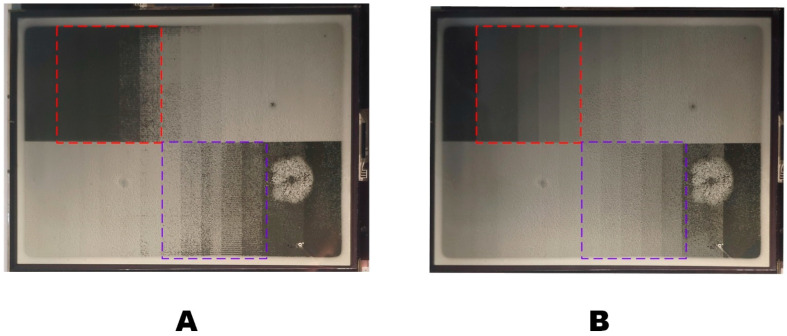
Each driving waveform was applied to a TFT-EWD to display gray scales, the red and purple squares show the difference of grayscale display under different waveforms. (**A**) The EWD driving by a 15 V DC driving waveform. (**B**) The EWD driving by a multi-DC driving waveform.

When the driving waveform was applied to the TFT-EWD, the parameters of the driving waveform could change by capacitance in the driving substrate of the EWD. Therefore, the driving waveform parameters in the TFT-EWD were set as follows: f = 62.06 Hz, $t_{2}$ = 61.38 us, $t_{3}$ = 9.29 ms, $V_{max}$ = 20 V, $V_{min}$ = 0 V, $V_{base}$ = 15 V. The driving waveform was taken as a common voltage and input into the top ITO substrate. The coordination of the source signal and the gate signal in the TFT substrate controls the voltage of the corresponding pixel of the EWD, thereby displaying the grayscale. As shown in Figure 16, each driving waveform was applied to the TFT-EWD to display multi levels of the gray scale. As could be seen in Figure 16A, pixels could not be driven when low gray scales were applied by a single DC driving waveform. As shown in Figure 16B, compared with the DC driving waveform, more gray scales could be displayed with a multi-DC driving waveform. As shown in Figure 16B, in the image of the area marked by the red box, the driving waveform proposed in this paper displays four more gray levels than the DC driving waveform. In the image of the area marked by the blue box, the grayscale image displayed more evenly and clearly with a proposed driving waveform.

**Figure 17:**
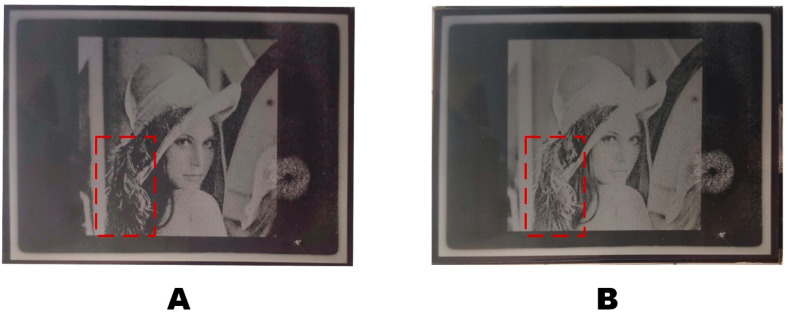
The picture of “Lena” displayed on the EWD, the red rectangles show the difference between the two different waveforms. (**A**) Display with the +15 V DC waveform. (**B**) Display with the proposed multi-DC waveform.

Compared with the DC driving waveform, the “Lena” image displayed by the multi-DC driving waveform proposed in this paper had richer details and greater brightness, as shown in Figure 17. Figure 17A showed the image driven by DC +15 V, and Figure 17B showed the image driven by the proposed waveform. As shown in Figure 17A, in the image of the area marked by the red box, the image was missing and dark with DC driving. As shown in Figure 17B, the overall brightness of the image was improved, and the dark details of the image were preserved because the content could be displayed with low voltage.

**Figure 18:**
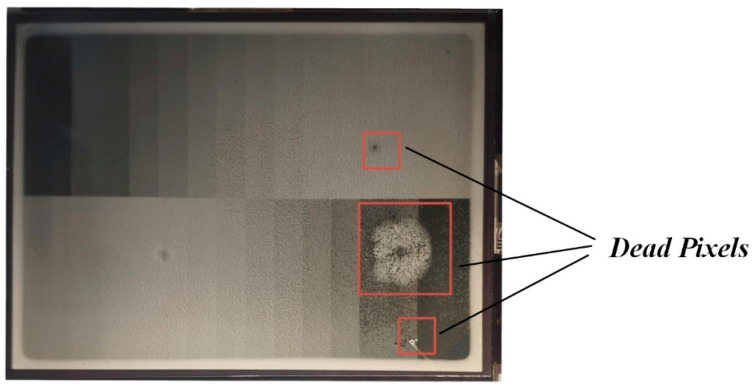
The EWD displays anomaly analysis.

As shown in Figure 18, some dead pixels occurred during the EWD test. It was mainly affected by the production process when dead pixels appear, which affect the display effect.

## 4. Conclusions

This paper designed a multi-DC overdriving waveform of an electrowetting display for gray scale consistency. The difference between the proposed driving waveform and the conventional driving waveform was that the square wave and overdriving wave were combined, which could not only inhibit the oil backflow but also realize a good gray scale consistency. In addition, to verify the effectiveness of the driving waveform, the driving waveform experiment platform was designed and ITO-EWD samples were used to study the waveform-related parameters. The driving waveform proposed in this paper has excellent gray scale consistency, and is a reference for the design of EWD driving methods.

## Figures and Tables

**Table 1:** The test parameters of the sample.

Sample Number	Process Conditions		Characteristic of Oil		Pixel Wall Height (µm)	Oil Droplet	Aperture Ratio (DC 30 V)
	Descum Parameter	Reflow Temperature	Oil Color	Concentration			
ITO-EWD	100 W/100 S	185 °C/2H	Black	18%	3.5	18	61.48%
TFT-EWD	100 W/100 S	210 °C/1H	Black	18%	3.5	18	\

## Data Availability

Data is contained within the article.
